# Dopamine Transporter maging with Tc-99m TRODAT-1 SPECT in Parkinson’s isease and its orrelation with linical isease everity

**DOI:** 10.22038/AOJNMB.2018.30356.1208

**Published:** 2019

**Authors:** Asra Patel, Shelley Simon, Indirani M Elangoven, Jaykanth Amalchandran, Avani S. Jain, Thangalakshmi S

**Affiliations:** Department of Nuclear Medicine and PET-CT, Apollo Hospitals, Chennai, India

**Keywords:** H and Y scale, Parkinson’s disease, Severity, Tc-99m TRODAT-1 SPECT, UPDRS

## Abstract

**Objective(s):**

To evaluate the role of Tc-99m TRODAT-1 Single Photon Emission Computed Tomography (SPECT) in Parkinson’s Disease (PD) by assessing the correlation of clinical disease severity, disease duration and age at onset of disease with specific uptake ratio of Tc-99m TRODAT-1 in striatum.

**Methods:**

The study included 63 patients in age range of 40-72 years with clinical diagnosis of PD and nine controls. Clinical history of patients was obtained regarding age at onset of disease and disease duration. Disease severity in each patient was assessed using H and Y stage and UPDRS. Tc-99m TRODAT-1 SPECT was performed and specific uptake ratios were calculated for six regions in bilateral striata, caudate nuclei and putamina. Difference in specific uptake ratios between different stages of disease was analyzed for statistical significance. Specific uptake ratios were correlated with UPDRS, motor score of UPDRS, duration of disease and age at onset of disease using Pearson’s correlation co-efficient.

**Results:**

Median specific uptake ratio was found to be least in contralateral putamen for all H and Y stages. There was a statistically significant difference between specific uptake ratios of controls vs stage 1, stage 1 vs 2, 1 vs 3, 1 vs 4, and 2 vs 4 for all 6 regions. The difference in uptake ratio between 3 and 4 H and Y stages was significant only for contralateral regions. There was no significant difference in uptake ratio between 2 and 3 H and Y stages. The uptake ratios showed a strong negative correlation with UPDRS and motor score, a weak negative correlation with duration of disease and no significant correlation with age at onset of disease.

**Conclusion:**

We conclude that Tc-99m TRODAT-1 SPECT can be used to assess the disease severity in PD patients.

## Introduction

Parkinson’s disease (PD), a chronic progressive neurodegenerative disorder, is mainly a disease of elderly with onset around fifth and sixth decades (1). Approximately 25% of patients clinically diagnosed to have PD, have an alternative diagnosis such as essential tremors (ET) and atypical Parkinsonian syndromes that mimic PD (2-6). It has been established by several studies that Dopamine Transporter (DAT) imaging with Tc-99m TRODAT-1 Single Photon Emission Computed Tomography (SPECT) has high sensitivity and specificity in differentiating PD from ET (3). PD follows a specific pattern on DAT SPECT showing greater reduction Tc-99m TRODAT-1 binding on the side contralateral to the symptomatic side and a gradient of decrease in tracer binding with greater reduction in putamen as compared to the caudate nucleus (2,7,8). 

Mixed results were obtained in different studies using different DAT ligands conducted to assess correlation between striatal binding ratios and disease severity, age and disease duration (2, 9-13). Most of the studies used tracers other than Tc-99m TRODAT-1 as the DAT binding agent to determine the correlation (9-12). Tc-99m TRODAT-1 has advantages of easy availability of Tc-99m, lower cost, optimal energy for imaging and faster pharmacokinetics allowing image visualization within hours. Hence, this study was conducted to assess if a similar correlation exists using Tc-99m TRODAT-1 as the DAT binding agent.

## Methods

The study included sixty three patients (42 males, 21 females) in age range 40-72 years, with clinical diagnosis of Parkinson’s disease, no identifiable secondary cause of Parkinsonism on Magnetic Resonance Imaging (MRI), and Tc-99m TRODAT-1 SPECT scan features suggestive of asymmetrical pattern of tracer uptake. Nine healthy controls (five male, four female) in age range of 58-70 years were also included. Informed consent was obtained from cases and controls. This study was approved by the ethical committee of the institute. 

**Figure 1:**
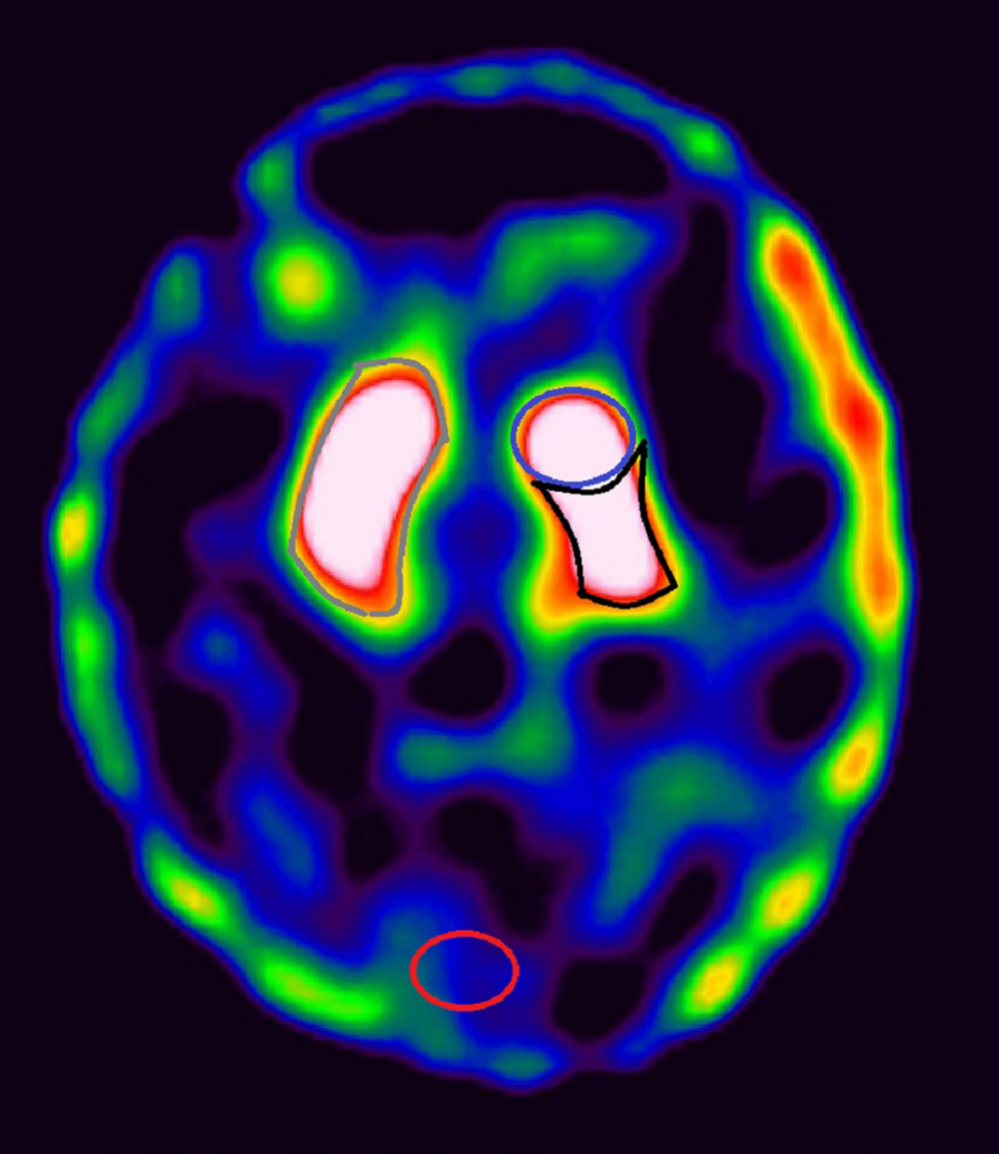
Image showing representation of manual ROI drawn over right striatum (grey), left caudate nucleus (blue), left putamen (black) and occipital region (red). Similar ROI were drawn over left striatum, right caudate nucleus and right putamen

Asymmetry of tracer uptake was assessed using Asymmetry Index calculated using the formula: ASI = [2×100× (Ipsilateral binding ratio – contralateral binding ratio)/ (Ipsilateral binding ratio + contralateral binding ratio)] (8). Patients with history or clinical examination suggestive of Major depressive disorder (MDD) were excluded from the study (14). Anti-Parkinsonian drugs were with-held 24 hours prior to the scheduled scan in order to avoid drug interference. A structured medical history was obtained and disease severity was assessed by using the Unified Parkinson’s Disease Rating Scale (UPDRS) and Hoehn and Yahr (H and Y) staging. Motor score was calculated using part three of UPDRS. After obtaining informed consent, 1110 MBq (30 mCi) of Tc-99m TRODAT-1 was administered intravenously. SPECT images were acquired three hours post injection using Symbia Siemens T6 dual head Gamma camera with patient in supine position and head positioned in a head holder, using acquisition parameters: zoom of 1.23, 128×128 matrix, 60 projections, and 30 seconds per projection. Image reconstruction was done using iterative reconstruction method. Six consecutive axial sections showing maximum uptake in the region of striatum were determined visually and were summed up (15). Regions of Interest (ROI) were drawn over bilateral striata, ipsilateral caudate nucleus, putamen, contralateral caudate nucleus, putamen (as shown in Figure 1) and specific uptake ratios were calculated for all six regions using occipital region as background on a summed up frame (15,16) as follows:

Specific uptake ratio = (counts in that region − occipital counts) ÷ occipital counts

For control population, right side was considered as ipsilateral side and left side was considered as contralateral side. 

All the continuous data was assessed for the normality using Shapiro Wilk’s test. As the variables did not follow normal distribution, they were expressed as median/Inter-quartile range. Comparison of variables was done by Mann Whitney U test. All the p values < 0.05 were considered as statistically significant. Specific uptake ratios were correlated with the clinical disease severity (H and Y staging, UPDRS and motor score), duration of disease and age at onset of disease using Pearson’s correlation coefficient (r). Data analysis was done using Statistical Package for Social Sciences (SSPS) version 11.0.

## Results

**Figure 2:**
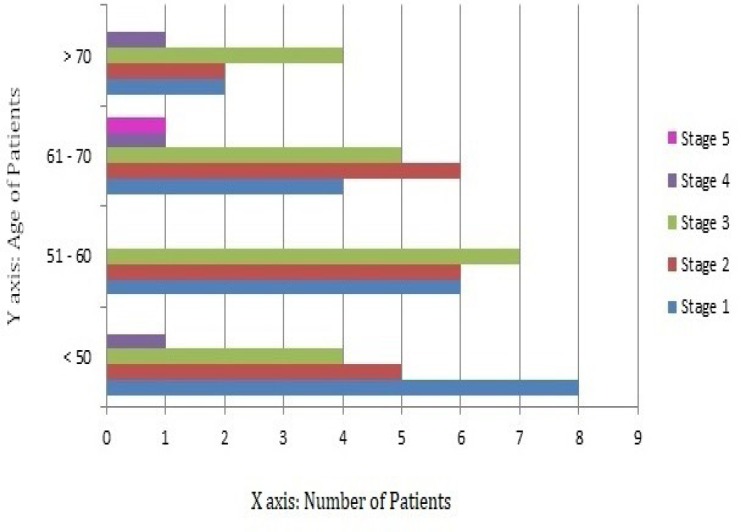
Horizontal bar diagram depicting distribution of study population in different age groups

Among the sixty three patients included in the study, twenty patients belonged to stage 1, nineteen patients to stage 2, twenty patients to stage 3, three patients to stage 4 and one patient to stage 5 of H and Y staging system. The distribution of study population according to age at onset of disease is provided in Figure 2. The median specific uptake ratio for stages 1, 2, 3 and 4 of H and Y scale (summarized in Table 1) was found to be least in the contralateral putamen followed by the ipsilateral putamen, contralateral caudate and ipsilateral caudate. The uptake ratio in the contralateral striatum was found to be lower than the ipsilateral striatum. As only one patient belonged to H and Y stage 5, the median could not be calculated. The difference between the specific uptake ratios of control group and case group was analyzed for statistical significance using Mann Whitney U test and was found to be statistically significant (p<0.001).

There was a difference in the uptake ratios of various H and Y stages of PD and was analyzed for statistical significance using Mann Whitney U test and Wilcoxon W test. The difference was statistically significant for all six regions between stages 1 vs 2, 1 vs 3, 1 vs 4 (<0.001), 2 vs 4 (<0.01 for contralateral and <0.03 for ipsilateral regions) and only for contralateral regions between stage 3 vs 4 (<0.03). The difference between stages 2 vs 3 was not significant for any of the six regions. 

**Figure 3:**
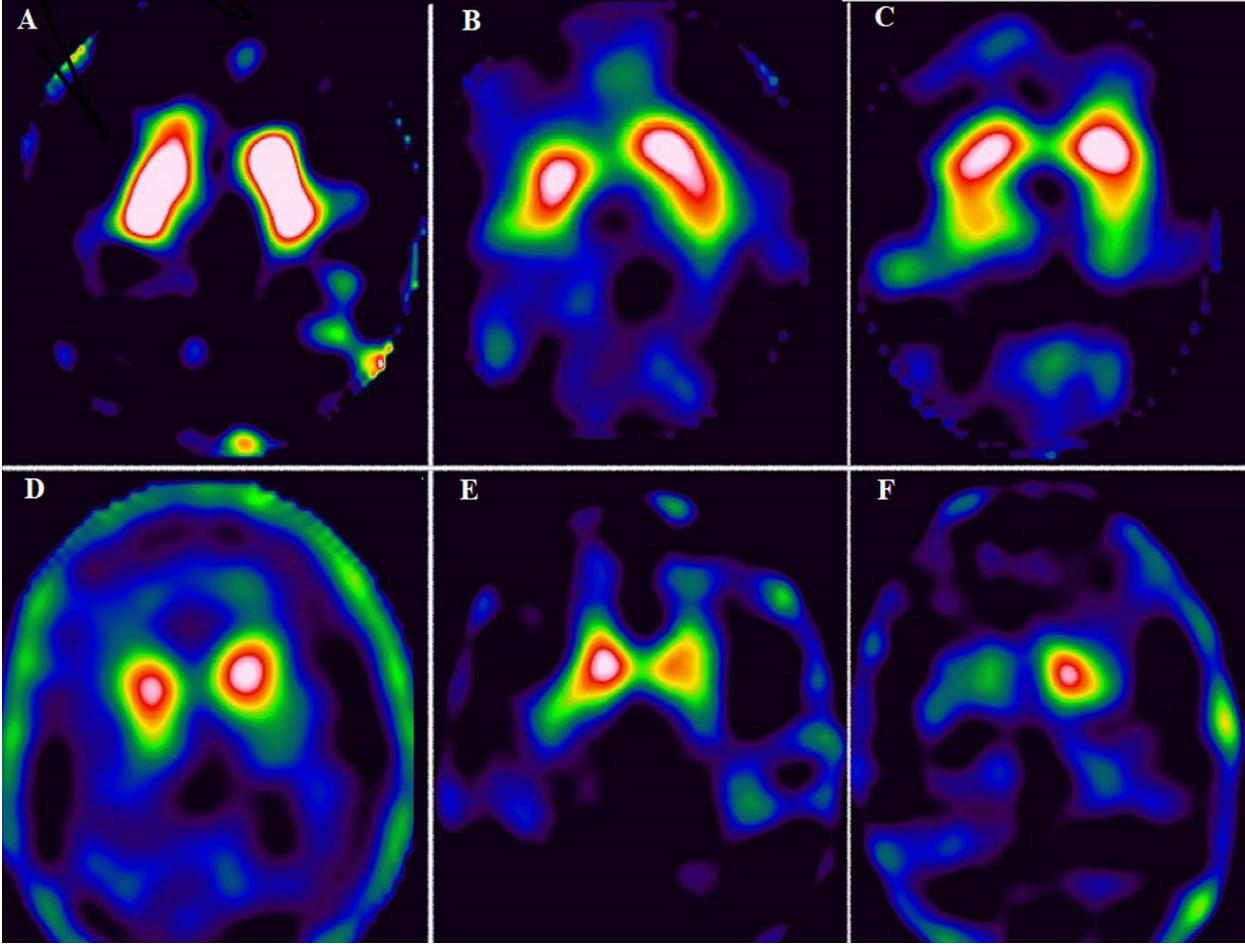
Tc-99m TRODAT-1 SPECT image of control (A) and patients belonging to different H and Y stages (B-F). B: stage 1, patient had history of rest tremors in left upper limb since 2 years; image shows reduced uptake in right putamen; C: stage 2, patient presented with difficulty in writing, rest tremor in bilateral upper limb, more on right side, image shows reduced ^99^^m^Tc-TRODAT-1 uptake in bilateral putamen (L<R) with preserved uptake in bilateral caudate nuclei; D: stage 3, patient had bilateral rest tremor and difficulty in walking; image shows no significant uptake in bilateral putamen, reduced uptake in right caudate nucleus. Uptake in left caudate nucleus is preserved; E: stage 4, patient presented with slowness of movements, rest tremors, difficulty in walking without support; image shows no significant uptake in bilateral putamen and reduced uptake in left caudate nucleus. Uptake in right caudate nucleus also appears reduced; F: stage 5, patient had rest tremors, was wheel chair bound, rigidity in all 4 limbs (R>L); image shows no significant uptake in bilateral putamen and right caudate nucleus, and reduced uptake in left caudate nucleus

Pearson’s Correlation coefficient (r) values for correlation between the specific uptake ratios in all 6 regions and UPDRS and motor score have been provided in Table 2. There was weak negative correlation between specific uptake ratios in all 6 regions and disease duration (<0.05) and no significant correlation with age at onset of disease. SPECT images of control and patients belonging to H and Y stages 1 to 5 have been provided in Figure 3 (A-F). 

**Table 1:** Median specific uptake ratios (with inter-quartile range)

**Population**	**Ipsilateral putamen**	**Contralateral putamen**	**Ipsilateral caudate**	**Contralateral caudate**	**Ipsilateral striatum**	**Contralateral striatum**
Control	9.23 (8.30-10.02)	9.15 (8.57-9.94)	5.70 (5.50-5.98)	5.84 (5.60-6.38)	17.37 (16.65-17.85)	17.25 (16.78-17.56)
H and Y Stage 1	3.81 (3.40-3.81)	3.49 (3.10-3.49)	4.01 (3.54-4.01)	3.89 (3.52-3.89)	8.77 (7.96-8.77)	7.42 (6.74-7.42)
Stage 2	2.17 (1.60-2.17)	1.96 (1.49-1.96)	2.77 (1.86-2.77)	2.46 (1.77-2.46)	5.94 (4.50-5.94)	4.48 (3.21-6.64)
Stage 3	2.14 (1.38-2.14)	1.81 (1.20-1.81)	2.83 (1.76-2.83)	2.43 (1.59-2.43)	5.98 (4.12-5.98)	4.28 (2.75-4.28)
Stage 4	1.72 (0.58-1.72)	1.22 (0.14-1.22)	1.89 (1.74-1.89)	1.67 (1.29-1.67)	4.61 (3.32-4.61)	2.89 (1.4-2.89)

**Table 2:** Correlation of specific uptake ratios in different regions of striatum with UPDRS score and Motor score

**Region**	**UPDRS Score**		**Motor Score**	
	**r value**	**P value**	**r value**	**P value**
Contralateral putamen	-0.912	0.001	-0.859	0.001
Ipsilateral putamen	-0.900	0.001	-0.848	0.001
Contralateral caudate	-0.902	0.001	-0.855	0.001
Ipsilateral caudate	-0.908	0.001	-0.854	0.001
Contralateral striatum	-0.910	0.001	-0.860	0.001
Ipsilateral striatum	-0.909	0.001	-0.857	0.001

## Discussion

In this study, we found a difference between the median specific uptake ratios in all six regions in H and Y stages of PD. The difference between stage 1 and 2, 1 and 3, 1 and 4, 2 and 4 were found to be statistically significant for all six regions. The difference between stages 3 and 4 was significant for the contralateral regions. The difference between stage 2 and 3 was not significant for any of the six regions. Clinically, both the stages (2 and 3) of H and Y scale have bilateral disease involvement. There is considerable overlap of disease symptoms in stages 2 and 3 – slowing of movements, posture and gait are affected in both. There is only minimal balance impairment in stage 3 disease (17). Although overall disease symptoms are considered to be mild in stage 2 and moderate in stage 3, there is a possibility of subjective variation in describing the severity of disease symptoms by the patient. This could explain the lack of significant difference between the stages 2 and 3. W S Huang et al. also reported a statistically significant difference in specific uptake ratios between stages 1 and 2. However they did not analyze the difference between other stages (16).

There was a statistically significant strong negative correlation between the specific uptake ratio and the UPDRS score. This correlation was strongest with the contralateral putamen followed by contralateral striatum. This was in concordance with study conducted by W S Huang et al which showed similar inverse correlation between the disease severities assessed using the H and Y staging (16). Booij et al also suggested that there is a significant negative correlation between specific uptake ratio of I-123 FP-CIT with H and Y staging. However, they did not find a significant correlation between the uptake ratios and motor score (18).

KV Laere et al conducted a study on two groups of patients, Tc-99m TRODAT-1 group and I-123 FP-CIT group. In the Tc-99m TRODAT-1 group, disease severity assessed using modified H and Y staging and specific uptake ratios in either putamina or caudate nuclei did not show significant correlation. However, in their study, patients in both the groups were continuing L-DOPA and dopamimetic agents during imaging. The number of patients on treatment during the imaging was higher in Tc-99m TRODAT-1 group as compared to the I-123 FP-CIT group (19). This raises a possibility of drug interference. Also, KV Laere et al included only patients with H and Y stage 1 and 2. This could explain the difference in results obtained in our study.

Our study also showed a strong negative statistically significant correlation between the specific uptake ratio and motor score. This correlation was strongest with contralateral striatum followed by contralateral putamen. Similar findings were reported in the study conducted by C S Lu et al using Tc-99m TRODAT-1 SPECT in patients with PD (20). Spiegel et al and W J Hwang et al also found significant inverse correlation between specific uptake ratios and motor score (9, 21). 

There was statistically significant weak negative correlation between the specific uptake ratio and the duration of the disease. This correlation was strongest with contralateral putamen followed by contralateral striatum and contralateral caudate. This finding is in agreement with Benamer et al, who also reported a significant correlation between mean specific uptake ratios and duration of disease (11). KV Laere et al found a significant correlation between disease duration of patients belonging to H and Y stage 1 and specific uptake ratio of putamen alone in I-123 FP-CIT Group. No correlation was seen between specific uptake ratios of any of the six regions with disease duration in Tc-99m TRODAT-1 group. The probable reasons for the difference in results could be that the mean disease duration in KV Laere et al study was low as compared to our study, it included only early PD patients and the patients in both the groups were on L-DOPA or dopamimetic agents (19). 

No significant correlation was obtained in our study between the specific uptake ratio in each of the region and age at onset. This is in concordance with the results obtained by Spiegel et al in their study using I-123 FP-CIT (9).

## Conclusion

Based on this study, we conclude that contralateral putamen was most severely affected in all stages of Parkinson’s disease. Specific uptake ratios decrease significantly with disease progression, increasing UPDRS score and motor score. Contralateral striatum is a better indicator of motor disease severity as compared to contralateral putamen. The specific uptake ratios also decrease with increase in disease duration. Thus Tc-99m TRODAT-1 SPECT can be used to assess the disease severity in PD patients. This can help the clinician in monitoring the disease progression, evaluating the therapeutic efficacy, predicting the disease prognosis and better patient counselling for further management.
